# Mechanical stimuli activate gene expression via a cell envelope stress sensing pathway

**DOI:** 10.1038/s41598-023-40897-w

**Published:** 2023-08-26

**Authors:** Christine E. Harper, Wenyao Zhang, Junsung Lee, Jung-Ho Shin, Megan R. Keller, Ellen van Wijngaarden, Emily Chou, Zhaohong Wang, Tobias Dörr, Peng Chen, Christopher J. Hernandez

**Affiliations:** 1 Sibley School of Mechanical and Aerospace Engineering, Cornell University, Ithaca, NY 14853 USA; 2 Meinig School of Biomedical Engineering, Cornell University, Ithaca, NY 14853 USA; 3 Department of Chemistry and Chemical Biology, Cornell University, Ithaca, NY 14853 USA; 4 Weill Institute for Cell and Molecular Biology, Cornell University, Ithaca, NY 14853 USA; 5 Department of Microbiology, Cornell University, Ithaca, NY 14853 USA; 6 Cornell Institute of Host-Microbe Interactions and Disease, Cornell University, Ithaca, NY 14853 USA; 7 Department of Bioengineering and Therapeutic Sciences and Orthopaedic Surgery, University of California, San Francisco, CA 94143 USA; 8 Chan Zuckerberg Biohub, San Francisco, CA 94158 USA

**Keywords:** Biomedical engineering, Mechanical engineering, Bacteria, Bacterial physiology

## Abstract

Mechanosensitive mechanisms are often used to sense damage to tissue structure, stimulating matrix synthesis and repair. While this kind of mechanoregulatory process is well recognized in eukaryotic systems, it is not known whether such a process occurs in bacteria. In *Vibrio cholerae*, antibiotic-induced damage to the load-bearing cell wall promotes increased signaling by the two-component system VxrAB, which stimulates cell wall synthesis. Here we show that changes in mechanical stress within the cell envelope are sufficient to stimulate VxrAB signaling in the absence of antibiotics. We applied mechanical forces to individual bacteria using three distinct loading modalities: extrusion loading within a microfluidic device, direct compression and hydrostatic pressure. In all cases, VxrAB signaling, as indicated by a fluorescent protein reporter, was increased in cells submitted to greater magnitudes of mechanical loading, hence diverse forms of mechanical stimuli activate VxrAB signaling. Reduction in cell envelope stiffness following removal of the endopeptidase ShyA led to large increases in cell envelope deformation and substantially increased VxrAB response, further supporting the responsiveness of VxrAB. Our findings demonstrate a mechanosensitive gene regulatory system in bacteria and suggest that mechanical signals may contribute to the regulation of cell wall homeostasis.

## Introduction

Mechanical forces have long been recognized as key contributors to the growth and function of organisms. In mammalian systems, mechanical forces regulate a wide variety of processes including cell differentiation during development^1,2^, disease initiation and progression^3^, and tissue homeostasis^4^. In tissues and organs with load-bearing functions, mechanical forces often act as the primary signal that initiates tissue remodeling and repair, thereby enabling the tissue to adapt to the mechanical challenges of the environment and quickly return to load bearing. Tissue remodeling thereby maintains homeostasis of mechanical function by balancing the removal of damaged tissue with tissue synthesis. Load-bearing structures including bone^5^, blood vessels^6^, and the plant cytoskeleton^7,8^ use mechanosensitive mechanisms to maintain mechanical function.

Most studies of mechanobiology focus on eukaryotic systems^9^, although recent evidence has highlighted the importance of mechanical forces in prokaryotes. In bacteria, extracellular appendages including flagella and type IV pili extend from the cell body to sense and respond to mechanical cues in the environment. Flagellar motor unit assembly and disassembly respond to increases and decreases in external mechanical load^10,11^. Physical inhibition of flagellar rotation by contact with a surface generates reaction forces within the molecular motor, which stimulate surface adhesion and biofilm formation^12,13^. Type IV pili are motorized fibers that extend and retract to interact with the environment. In addition, mechanosensing by Type IV pili promotes biofilm formation^14^ and the release of virulence factors^15^, and guides motility after collisions^16^.

The cell envelope is the primary load-bearing component of bacteria and is also sensitive to mechanical forces. Stretch-activated ion channels within the cell membrane rapidly respond to changes in osmolarity by opening due to membrane stretching, leading to increased survival following hypo-osmotic shock^17^. Mechanical stress and strain within the cell envelope also affect the assembly of trans-envelope efflux complexes; for example, assembly and function of the trans-envelope multicomponent efflux pump CusCBA is impaired by increases in octahedral shear stress within the cell envelope^18^. Mechanical stress within the cell envelope also affects the locations of insertion of new cell wall in bacteria submitted to bending, with greater amounts of cell wall inserted at regions of greater tensile strain^19,20^. Although these mechanosensitive mechanisms within the cell envelope are well recognized, none of the mechanisms identified to date have been shown to regulate gene expression related to the remodeling of the cell wall, an essential component of the cell envelope. It has been hypothesized that gene regulatory systems currently associated with other forms of cell stress may also be mechanosensitive^21^ and a recent report suggests that confinement enhances Rcs signaling and subsequent resistance to bacteriophage in *E. coli*^22^. If mechanosensitive mechanisms are involved in remodeling and homeostasis of the cell envelope, mechanical stress and strain would be expected to regulate the synthesis of components of the cell envelope.

*Vibrio cholerae*, the causative agent of cholera disease, survives rapid changes in osmolarity during the transition between fresh and brackish water, marine environments, and the intestines of a host. Osmolarity changes result in fluctuations in turgor that alter mechanical stress in the cell envelope. The primary structures counteracting turgor are the peptidoglycan (PG) cell wall and the outer membrane (OM)^23^. To maintain adequate mechanical properties, bacteria must therefore maintain mechanical integrity of both PG and OM, and this indeed seems to be the case for the OM^23,24^. If and how PG strength is homeostatically controlled is poorly understood.

**Figure 1:**
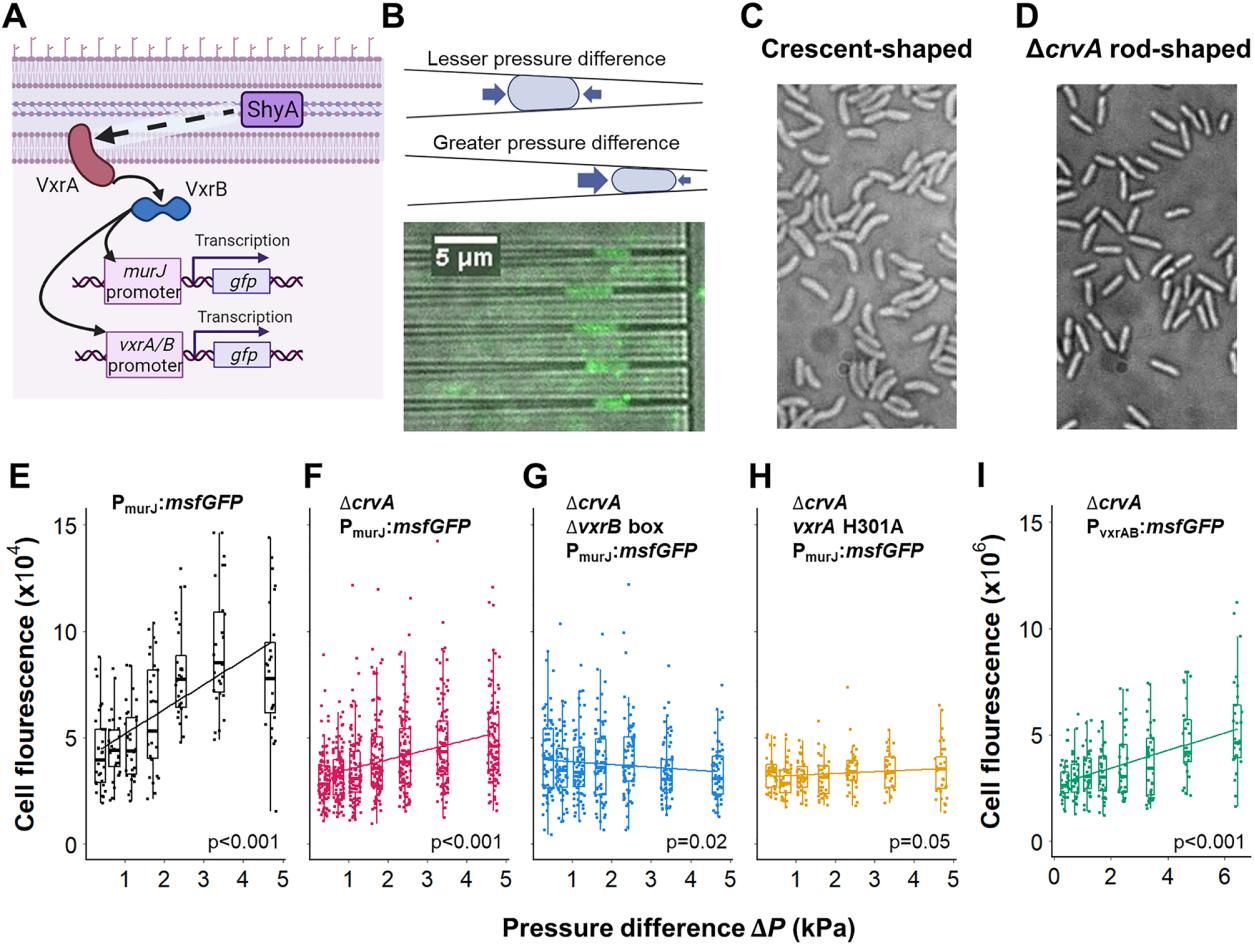
VxrAB signaling responds to mechanical stress from extrusion loading. (**A**) When activated, inner membrane histidine kinase VxrA phosphorylates response regulator VxrB, which regulates gene expression of regulons that include *murJ* and *vxrAB*. Transcriptional *msfGFP* fusions for *murJ* and *vxrAB* were used as reporters for VxrAB signaling. ShyA function promotes VxrA signaling through an unknown mechanism. (**B**) Extrusion loading involves forcing bacteria under fluid pressure into tapered channels. (top) Cells deform more and experience greater mechanical loading at a greater pressure difference in tapered channels. (bottom) Δ*crvA* P_murJ_:*msfGFP Vibrio cholerae* expressing GFP while trapped within tapered channels are shown (a "set" of tapered channels with the same differential pressure is shown). (**C**) *Vibrio cholerae* are crescent-shaped. (**D**) *Vibrio cholerae* with *crvA* deletion are rod-shaped. (**E**) Single cell fluorescence of P_murJ_:*msfGFP* cells vs pressure difference. Solid line is a linear regression. (**F**) Single cell fluorescence of Δ*crvA* P_murJ_:*msfGFP* cells (pink) and (**G**) Δ*crvA* Δ*vxrB* box P_murJ_:*msfGFP* cells (blue) vs pressure difference. Solid lines are linear regressions. Slope of Δ*crvA* P_murJ_:*msfGFP* cell (pink) is greater than the slope of the Δ*crvA* Δ*vxrB* box P_murJ_:*msfGFP* cells (blue) (p < 0.001). (**H**) The *vxrA* H301A mutant with modified histidine kinase. (**I**) Single cell fluorescence of Δ*crvA* P_vxrAB_:*msfGFP* cells vs pressure difference. Solid line is a linear regression.

In *Vibrio cholerae,* the VxrAB two-component system is the major cell wall stress response system^25–27^. VxrAB is induced by exposure to cell wall-acting antibiotics and overexpression of cell wall lytic enzymes within the periplasm like the endopeptidase ShyA^27,28^. Upon induction, VxrAB upregulates its own expression, as well as cell wall synthesis functions, including the PG translocase MurJ, the major PG synthases (penicillin-binding proteins, PBPs), and PG precursor synthesis genes (Fig. 1A)^27^. Consequently, VxrAB activation results in increased cell wall content and enhanced resistance to osmotic shock^27^. These mechanisms are consistent with the idea that VxrAB contributes to cell wall homeostasis, similar to WalKR in the Gram-positive bacterium *Bacillus subtilis*^29^*.* Consistent with this idea, a ∆*vxrAB* mutant exhibits increased cell width during normal growth^27^, a finding expected if cell wall stiffness were impaired but turgor pressure remained the same. Importantly, VxrAB is also essential for survival after exposure to cell wall-acting antibiotics such as beta-lactams^27,28^. Beta-lactam exposure induces large-scale alterations of cell envelope mechanical properties, including a change from a rigid rod-shaped cell contained by a cell wall, to a membranous spheroplast^30,31^. Recovery from the spheroplast state relies primarily on VxrAB^28^, presumably since rod shape regeneration requires increased cell wall synthesis. Thus, the VxrAB system modulates the mechanical properties of the cell in response to imbalances in PG turnover. The signal resulting in VxrAB induction, however, is still unknown.

Here we study VxrAB controlled gene expression during mechanical stimulation of the cell envelope of *Vibrio cholerae*. We applied mechanical stress and strain to the bacterial cell envelope using two distinct loading modalities: extrusion loading within a microfluidic device, whole cell compression, and hydrostatic pressure. In each of these situations we find that bacteria experiencing greater magnitudes of mechanical loading exhibit greater VxrAB regulon activation. Our findings support the idea that mechanical stress and strain play a role in regulating cell envelope synthesis and homeostasis and demonstrate the existence of gene regulatory systems that can be induced by mechanical stress in the cell envelope.

## Results and discussion

### VxrAB signaling is activated by extrusion loading

To investigate the role of mechanical stress on VxrAB signaling, we used a custom microfluidic device to apply controlled, reproducible mechanical loads in a process we call “extrusion loading”. Extrusion loading uses fluid pressure to push bacteria into narrow tapered channels with sub-micron dimensions (Fig. 1B)^18,32^. Cells become lodged in the tapered channels and experience mechanical forces as they are deformed by the channel walls. The pressure difference (∆*P*) across the tapered channel regulates the magnitude of mechanical stress experienced by a trapped cell. Cells submitted to greater pressure difference inside the tapered channels travel further into the tapered channels and experience greater deformation by the channel walls and greater magnitudes of mechanical stress. Analytical and finite element models indicate that extrusion loading results in increases in axial tensile stress (along the length of a rod-like cell), reductions in hoop tensile stress (circumferentially around the cell envelope), and increases in octahedral shear (shape-changing) stress in the cell envelope^18^.

We used a transcriptional P_murJ_:*msfGFP* fusion as a well-established reporter for VxrAB signaling (Fig. 1A). MurJ encodes for lipid II flippase for peptidoglycan precursor, and flipping the peptidoglycan precursor into the periplasmic space is critical for cell wall assembly^33^. The response regulator VxrB has a high affinity for direct binding of the murJ promoter^28^, and *murJ* expression is consequently strongly controlled by VxrAB^27^, rendering this construct a robust readout of VxrAB activation. We applied extrusion loading to P_murJ_:*msfGFP* cells for two hours to allow sufficient time for transcription and protein folding, then measured the fluorescence of individual cells to quantify the msfGFP expression under MurJ promoter control. The fluorescence of P_murJ_:*msfGFP* cells increased with increasing magnitude of extrusion loading (Fig. 1E), supporting the idea that VxrAB signaling is mechanosensitive (although it is possible that the fluorescence saturates after a differential pressure of 3 kPa).

*Vibrio cholerae* cells naturally exhibit a crescent shape (Fig. 1C). The straightening of a crescent-shaped cell inside the microfluidic device during extrusion loading results in additional mechanical stresses including greater tensile stresses on the concave side of the cell and compressive stresses on the convex side. To determine whether the mechanosensitive fluorescent response was solely due to the stresses caused by cell straightening, we created rod-shaped *V. cholerae* by deleting *crvA* (Fig. 1D)^34^ and submitted Δ*crvA* P_murJ_:*msfGFP* cells to extrusion loading. CrvA is a periplasmic polymer protein that is responsible for curvature in *V. cholerae*; removal of crvA results in rod-shaped cells^34^. The fluorescence of Δ*crvA* P_murJ_:*msfGFP* cells also increased with increasing pressure difference (Fig. 1F, magenta), suggesting that cell curvature is not necessary for the mechanosensitive fluorescent response of P_murJ_:*msfGFP* cells during extrusion loading. The difference in slope of the fluorescence vs pressure difference between the crescent-shaped and the rod-shaped cells may be explained by the additional stresses that crescent-shaped cells experience from straightening. All subsequent experiments were performed with rod-shaped, Δ*crvA* cells. The autofluorescence signal of Δ*crvA* non-GFP producing cells was not increased at greater magnitudes of extrusion loading (Supplementary Fig. S1) nor was the reporter fluorescence related to magnitude of mechanical stress in the first minutes after the initiation of extrusion loading (Supplementary Fig. S2).

To confirm that the mechanosensitive response was due to VxrB-activated expression of P_murJ_:*msfGFP*, we used a mutant with a partial deletion of the VxrB binding site on the MurJ promoter (Δ*crvA* P_murJ_^Δ*vxrB* box^:*msfGFP*); we previously established that this mutant indeed lacks VxrAB-responsiveness^28^. Under extrusion loading, the fluorescence of such mutant cells showed only small, negative correlations with magnitude of pressure (Fig. 1G, blue). Cells deficient in *vxrAB* do not show a mechanosensitive response (Supplementary Fig. S3). Similarly, a mutation in the conserved phosphorylated histidine residue in VxrA in its native chromosomal locus (H301A strain) that results in a phenocopy of a ∆*vxrAB* strain (Supplementary Fig. S4) leads to a poor relationship between mechanical loading and P_murJ_ signaling (Fig. 1H, p = 0.05). Taken together, these data demonstrate that VxrB activation through the canonical histidine kinase function is required for the mechanosensitive increase in P_murJ_:*msfGFP* expression.

In addition to its role in MurJ induction, VxrAB signaling is also autoregulated (Fig. 1A)^28^. To confirm that the mechanosensitive response of VxrAB was not limited to the MurJ promoter, we also used a P_*vxrAB*_*:msfGFP* reporter strain as an alternative readout of VxrAB activation. Cell fluorescence of P_*vxrAB*_*:msfGFP* cells increased at greater pressure difference (Fig. 1I), confirming that mechanical stress in the cell envelope regulates VxrAB signaling (note that the basal activity of the VxrAB promoter differs from that of MurJ promoter resulting in different total amounts of cell fluorescence). We conclude that VxrAB activation is sensitive to mechanical stress from extrusion loading.

### VxrAB signaling is activated by compression

To confirm that the mechanosensitive response of VxrAB signaling is not specific to the microfluidic device environment or the forms of mechanical stresses generated by extrusion loading, we investigated the response of VxrAB signaling to other methods of mechanical loading.

**Figure 2:**
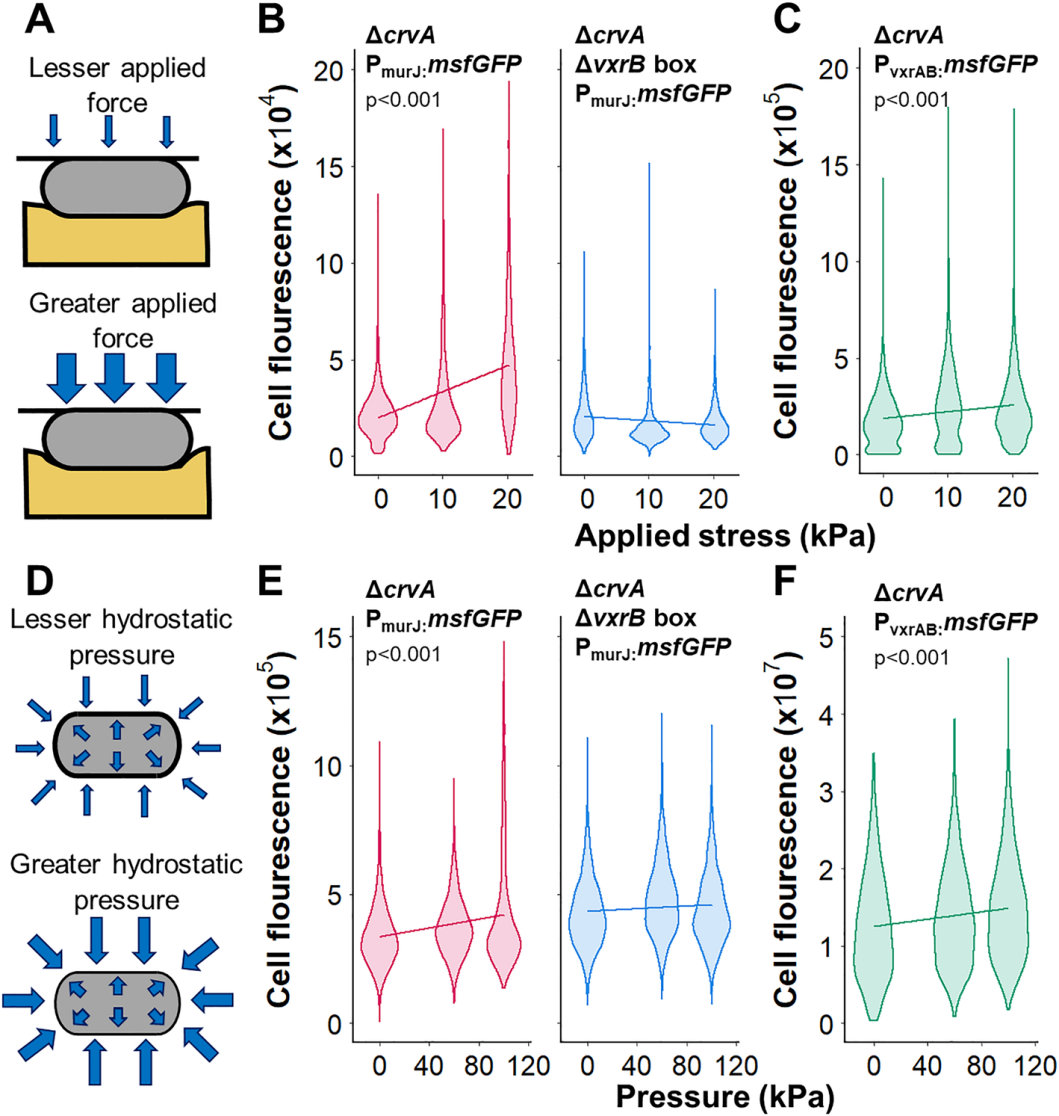
VxrAB signaling responds to mechanical stress from compression and hydrostatic pressure. (**A**) During compression, cells are sandwiched between agarose gel and a weighted glass slide. (**B**) Single cell fluorescence of Δ*crvA* P_murJ_:*msfGFP* cells (pink, n = 1130, 1160, 1304 in order of loading), Δ*crvA* Δ*vxrB* box P_murJ_:*msfGFP* cells (blue, n = 1461, 1189, 1304), and (**C**) Δ*crvA* P_vxrAB_:*msfGFP* cells (green, n = 1541, 1058, 1826) vs applied compression force. Solid lines are linear regressions. The slope of Δ*crvA* P_murJ_:*msfGFP* cells (pink) is greater than the slope of the Δ*crvA* Δ*vxrB* box P_murJ_:*msfGFP* cells (blue) (p < 0.001). (**D**) Hydrostatic pressure applies force equally to all surfaces of the cell compressing the cell envelope. (**E**) Single cell fluorescence of Δ*crvA* P_murJ_:*msfGFP* cells (pink, n = 3845, 2261, 1393), Δ*crvA* Δ*vxrB* box P_murJ_:*msfGFP* cells (blue, n = 2085, 1194, 1554). The slope of Δ*crvA* P_murJ_:*msfGFP* cells (pink) is greater than the slope of the Δ*crvA* Δ*vxrB* box P_murJ_:*msfGFP* cells (blue) (p < 0.001). (**F**) Δ*crvA* P_vxrAB_:*msfGFP* cells (green, n = 2332, 1971, 907) vs hydrostatic pressure. Solid lines are linear regressions.

To apply compression, cells were sandwiched between agarose gel and a weighted glass slide (Fig. 2A). At greater magnitudes of applied force, cells experienced greater mechanical loading and deformed to a greater cell width in the plane of imaging (Supplementary Fig. S5). Compression causes increases in tensile stress in the axial and hoop directions and overall increase in octahedral shear (shape-changing) stress within the cell envelope. P_murJ_:*msfGFP* cells were submitted to compression loading for two hours (the same duration that cells experienced under extrusion loading), and the fluorescence of individual cells was then measured to quantify expression of P_murJ_:*msfGFP*. Although there was greater variability in cell fluorescence, on average cell fluorescence increased 30% with increasing magnitude of applied compression force (Fig. 2B, left), indicating that VxrAB signaling is sensitive to compression loading. In contrast, upon deleting the *vxrB* box in the promoter (Δ*vxrB* box), the relationship between cell fluorescence and applied compression force was not detectable (Fig. 2B, right), suggesting that the mechanosensitive increase in cell fluorescence for P_murJ_:*msfGFP* cells under compression also required VxrB activation. We again used P_*vxrAB*_*:msfGFP* cells as a secondary reporter for VxrAB signaling. Cell fluorescence of P_*vxrAB*_*:msfGFP* cells increased with increasing applied compression force (Fig. 2C), further supporting the idea that the mechanosensitive response to compression is mediated by VxrAB. We note that msfGFP fluorescence shows larger variance among individual cells during compression loading than during extrusion loading, which is likely because this loading modality does not rigorously control the cell orientation relative to loading, the amount of load applied to each individual, or cell–cell contact, all of which may alter the mechanical stress state within the cell envelope.

To supplement these findings, we assessed the role of hydrostatic pressure in VxrAB induction. Hydrostatic pressure exerts force perpendicular to all surfaces of the cell envelope (Fig. 2D–F). Extreme hydrostatic pressure (> 50 MPa) causes changes to RNA synthesis and DNA replication and can cause cell death^35^; here we apply mild hydrostatic pressure of 0–100 kPa (a bacterium 10 m underwater experiences approximately 100 kPa hydrostatic pressure), in contrast a cell leaving a host gastrointestinal system and entering brackish water is expected to experience an increase in turgor pressure of 500 kPa. Hydrostatic pressure increases the compressive stresses perpendicular to the cell envelope. The magnitude of mechanical stresses caused by hydrostatic pressure are typically much smaller than the hoop and longitudinal stresses (oriented parallel to the cell envelope surface) generated by extrusion loading or compression, but under conditions of applied hydrostatic pressure can become noticeable. Hydrostatic pressure was applied to cells suspended in liquid media in a custom chamber connected to a microfluidic pump. After 2 h of incubation at room temperature, cells were removed and visualized. Average cell fluorescence increased 28% with increasing magnitude of applied compression force (Fig. 2E, left), supporting the idea that VxrAB signaling is also sensitive to hydrostatic pressure. Upon deleting the *vxrB* box in the promoter, cell fluorescence did not vary with applied hydrostatic pressure (Fig. 2E, right), confirming that the mechanosensitive increase in cell fluorescence for P_murJ_:*msfGFP* cells required VxrB activation. As in the compression experiments, the P_vxrAB_:*msfGFP* reporter strain were consistent with the observations with P_murJ_:*msfGFP* (Fig. 2F). The variance in fluorescence was greater than that seen in compression loading, a finding we attribute to the fact that mechanical stresses in the cell envelope are limited to radial compression and are not as well controlled (cell contact with the walls of the device and other cells is completely uncontrolled). As a result, the mechanosensitive response to hydrostatic pressure was dominated by a subset of cells (upper portion of cell population in Fig. 2E,F). We conclude that VxrAB signaling is responsive to diverse methods of applying mechanical load to the cell envelope.

### Cell wall turnover affects mechanosensitivity of VxrAB signaling

VxrAB is known to respond to cell wall damage caused by cell wall targeting antibiotics and to the activity of the endopeptidase ShyA through an unknown mechanism (Fig. 1A)^27^. The endopeptidase ShyA plays a key role in cell wall homeostasis and cell elongation through controlled cell wall degradation in *V. cholerae*^36^. Removal of ShyA may therefore modulate cell envelope stiffness, which may affect VxrAB and the associated mechanosensory response. To explore the possibility that VxrAB mechanosensitive signaling is dependent on upstream signals from ShyA, we exposed mutants in which *shyA* was deleted to extrusion loading and measured the resulting changes in VxrAB signaling.

**Figure 3:**
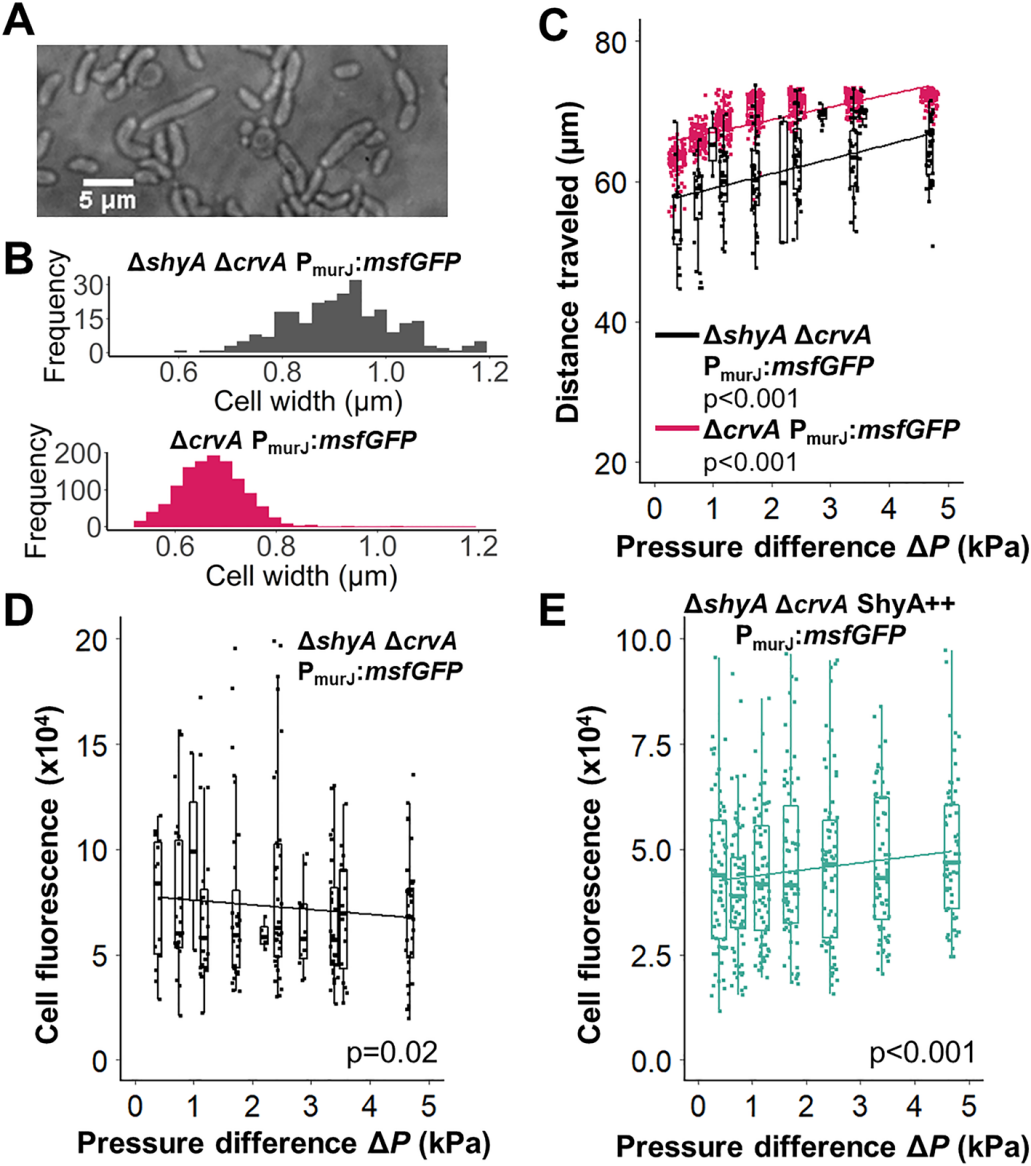
ShyA and VxrAB mechanosensitivity. (**A**) An image of Δ*shyA* Δ*crvA* P_murJ_:*msfGFP* cell morphology grown in M9 medium in a petri dish. Cells show abnormal morphology. (**B**) *Top*: Δ*shyA* Δ*crvA* P_murJ_:*msfGFP* cell width in a petri dish. *Bottom*: Δ*crvA* P_murJ_:*msfGFP* cell width in a petri dish. (**C**) Distance traveled in the tapered channel for Δ*shyA* Δ*crvA* P_murJ_:*msfGFP* cells (black) is less than in Δ*crvA* P_murJ_:*msfGFP* cells (pink) at similar pressure magnitudes. (**D**) Single cell fluorescence of Δ*shyA* Δ*crvA* P_vxrAB_:*msfGFP* cells vs pressure difference in extrusion loading (black). (**E**) Single cell fluorescence of Δ*shyA* Δ*crvA* ShyA +  + P_murJ_:*msfGFP* cells vs pressure difference shows a slope (2216 ± 613, estimate ± SE) closer to that in Fig. 1F (4690 ± 486). Solid green line is a linear regression.

Indeed, Δ*shyA* P_murJ_:*msfGFP* cells exhibited noticeably perturbed physiology with blebs, curves, and abnormal shape, suggesting the possibility of impaired cell envelope mechanical properties that might reduce cell stiffness (Fig. 3A). Cells with *shyA* deleted were wider than cells with normal *shyA* expression outside of the microfluidic device (Fig. 3B, top vs. bottom), an observation expected if the stiffness of the cell envelope were reduced. We speculate that deletion of ShyA may lead to reductions in the stiffness of the cell envelope, for example by causing overexpression of other cell wall lytic enzymes. ShyA deficient cells did not travel as far into the tapered channels during extrusion loading as cells with normal ShyA expression (Fig. 3C), potentially suggesting that cells defective in major endopeptidase activity experienced insufficient cell deformation compared with cells with normal ShyA expression. However, because they are initially wider, Δ*shyA* P_murJ_:*msfGFP* cells actually experience greater mechanical strain/deformation than the P_murJ_:*msfGFP* cells despite not traveling as far in the tapered channel. Indeed, when comparing cell width inside and outside the tapered channels, Δ*shyA* P_murJ_:*msfGFP* cells experienced a greater percentage decrease (average of 40% decrease, 0.91 ± 0.11 μm undeformed v. 0.54 ± 0.09 μm loaded, mean ± SD) in cell width as compared to P_murJ_:*msfGFP* cells (average of 25% decrease in cell width, 0.67 ± 0.06 μm undeformed v. 0.51 ± 0.03 μm loaded). Larger deformations within the tapered channels would be expected to enhance the response of mechanisms sensitive to mechanical strain within the cell envelope.

Consistent with this possibility, the baseline fluorescence of cells with *shyA* deleted (Δ*shyA* P_murJ_:*msfGFP,* 8.84e + 04 on average, Fig. 3D) under extrusion loading was greater than that in cells with intact *shyA* (4.04e + 04 on average, Fig. 1F). However, the response was poorly correlated with the magnitude of extrusion loading (a slight negative relationship rather than positive, Fig. 3D). When ShyA was reintroduced in trans (Δ*shyA* ShyA +  + P_murJ_:*msfGFP)*, the mechanosensitive response was partially restored: cell fluorescence increased with increasing pressure difference with a slope closer to that seen in the Δ*crvA* P_murJ_:*msfGFP* (Fig. 3E, compared to pink line in Fig. 1F). Since cells in tapered channels for both groups experience the same pressure difference, the poor correlation between fluorescence and pressure difference in the Δ*shyA* P_murJ_:*msfGFP* cells is not due to differences in applied mechanical force. Instead we interpret this finding to be very high, perhaps saturating, induction of the VxrAB signaling under the greater magnitudes of deformation experienced by Δ*shyA* cells. An additional possibility is that the absence of ShyA results in changes in PG structure in a way that makes it non-conducive to VxrAB-mediated mechanosensing. Although the mechanotransduction mechanisms that make VxrAB mechanosensitive remain unclear, these findings demonstrate how cell envelope stiffness can regulate the response of VxrAB signaling to mechanical loading.

## Conclusions

We have demonstrated that the two-component signaling system VxrAB is activated by mechanical stress in the cell envelope caused by distinct mechanical loading modalities. These findings indicate that mechanosensitive mechanisms within the cell envelope can regulate gene expression involved in cell wall remodeling.

Our findings regarding mechanosensitivity of VxrAB are consistent with the idea that mechanical stress and strain may contribute to cell wall homeostasis. The ability of mechanical stress and strain to contribute to the remodeling of a load-bearing component such as the cell wall is a powerful means of maintaining the function of the cell envelope. While we are unaware of other studies directly investigating the effects of mechanical stress on cell wall maintenance in other bacteria, there are other two-component systems that regulate cell wall remodeling. The two component system WalKR in *Bacillus subtilis* responds to degradation of cell wall constituents to regulate cell wall remodeling^29^. Additionally, recent findings indicate that the outer membrane is also capable of carrying mechanical loads^23^, opening the possibility that the synthesis and transport of outer membrane components may be sensitive to mechanical stress.

One limitation of this study is the variance in fluorescence response among cells at each applied load magnitude. The magnitude of variance follows the same pattern as the degree of control of mechanical loading of each cell (most control in the microfluidic system, less in compression loading, even less in hydrostatic pressure), suggesting that variation in mechanical stresses experienced by individual cells is the primary cause. Even with the most controlled loading system it is likely that there remains variance from cell to cell that may be caused by cell physiology or natural variance in cell envelope stiffness within the population. Further study would be required to understand the sources of this variance in more detail. Despite this limitation, the large number of cells examined makes it possible to detect meaningful trends relating applied mechanical load to VxrAB signaling.

The mechanosensitive nature of VxrAB suggests potential applications in the field of synthetic biology. Gene regulatory mechanisms that respond to the external environment are key tools in the field of synthetic biology. Systems that respond to target chemicals, light, temperature, and pH have been used to control synthetic gene circuits^37^. Our work demonstrates that two-component systems can respond to mechanical stress in the cell envelope, providing an additional mechanical mechanism for stimulating gene circuits for synthetic biology applications.

## Methods

### Microfluidic device manufacturing

The methods for microfluidic device manufacturing used here are similar to what is described in our previously published works^18,32^ except the etch depth was reduced to accommodate the smaller cell dimensions of *Vibrio cholerae* as compared to *E. coli* examined in prior work. Fused silica wafers (100 mm diameter and 500 µm thick, WF3937X02031190, Mark Optics, Santa Ana, CA, USA) were patterned using Deep UV photolithography in the cleanroom facility at Cornell NanoScale Facility Science and Technology Facility (Ithaca, NY, USA). Clean fused silica wafers were first coated with ~ 55 nm of chrome using the AJA Sputter Deposition Tool (AJA International, Scituate MA, USA). The Gamma Automatic Coat-Develop Tool (Suss MicroTec Gamma Cluster Tool, Garching Germany) was then used to apply a ~ 60 nm coat of anti-reflective coating (ARC, DUV 42P, Brewer Science, Rolla, MO, USA) and ~ 510 nm coat of photoresist (UV210, MicroChem, Westborough, MA, USA). The photoresist was exposed to our custom microfluidic device pattern using the ASML Deep UV stepper (Veldhoven Netherlands), then the photoresist was immediately developed using the Gamma Automatic Coat-Develop Tool. The pattern was transferred from the photoresist to the anti-reflective coating using plasma etching in the Oxford 82 Tool (Oxford, Abingdon, UK), then transferred to the chrome layer with plasma etching using the Plasma-Therm 770 ICP tool (Plasma-Therm St. Petersburg FL, USA). Any remaining anti-reflective coating was removed with a plasma oxygen clean in the Oxford 82 Tool. Finally, the pattern was transferred to the fused silica with plasma etching using the Oxford 100 Tool (Oxford, Abingdon, UK). The remaining chrome was removed using a wet chemical bath. Through-holes were laser-etched at the microfluidic device inlets and outlets using a Versalaser (VLS3.50, Universal Laser Systems, Scottsdale, AZ, USA).

The wafer feature dimensions were characterized using a profilometer, an atomic force microscope, and a scanning electron microscope. The target etch depth for the channels was at least two standard deviations greater than the cell width (> 0.80 µm) so the cells could flow freely through all of the feeder channels and would only get stuck in the narrow constriction of the tapered channels. The feeder channel etch depth was characterized using a profilometer (P-7, KLA Inc, Milpitas CA, USA). The profilometer tip was too wide to measure the tapered channels, so an atomic force microscopy high aspect ratio tip was used to measure the etch depth of tapered channels (Veeco Icon Bruker, Billerica MA, USA). Channel etch depth was 0.88 ± 0.03 µm. A scanning electron microscope (Zeiss Ultra 55 SEM microscope, Oberkocken Germany) was used to measure the tapered channel inlet width (1.48 ± 0.07 µm) and outlet width (0.35 ± 0.02 µm). The tapered channel inlet is wide enough that cells can enter, and the outlet is narrow enough to prevent cells from flowing out.

The patterned wafers were bonded to 100 mm diameter and 170 µm thick fused silica cover wafers (WF3937X0073119B Mark Optics, Santa Ana CA, USA). Cover wafer thickness was chosen to be the same thickness as standard coverslips. Both the patterned wafers and the cover wafers were MOS/RCA cleaned before bonding. Wafers were gently hand bonded, then underwent nitrogen annealing for 5 h at 1100 °C. Wafers were allowed to sit at least one week before use to let the bond mature.

### Microfluidic device design

The microfluidic device design has been described previously in our published works^18,32^. Fluid pressure pushes bacterial cells into narrow tapered channels, a process we refer to as extrusion loading (Supplementary Fig. S6). The cell experiences deformation and mechanical stress and strain in the cell envelope as it is constricted by the channel walls. The fluidic pressure is higher at the wide inlet of the channel and lower at the narrow outlet of the channel, pushing the cells towards the outlet. We refer to the difference in pressure between the wide inlet and the narrow outlet of the tapered channel as the pressure differential (ΔP). Cells in tapers with a greater pressure differential experience a greater magnitude of mechanical loading, travel further into the tapered channel, and deform more than cells in tapers with a lesser pressure differential. The distance the cell travels into the tapered channel is dependent on the magnitude of the pressure differential, initial cell width, cell stiffness and cell width prior to entrance into the tapered channel.

Hundreds of cells under multiple loading magnitudes are observed during each experiment. Twelve sets of five tapered channels are put in parallel and connected by a bypass channel (Supplementary Fig. S6). Pressure is highest at the inlet of the bypass channel and lowest at the outlet of the bypass channel due to pressure loss from hydraulic resistance; therefore, the tapered channels near the bypass inlet and outlet experience the greatest pressure differential (ΔP) (Supplementary Fig. S6). Ten bypass channels are put in parallel and connected by feeder channels to a single entry port for the microfluidic device (Supplementary Fig. S6). While trapped in the tapered channels cells remain alive for hours and are observed elongating and dividing.

### Loading cells into the microfluidic device

Fluid pressure was used to load the cells into the microfluidic device. Pressure was generated using a PneuWave Pump (CorSolutions, Ithaca NY, USA). PEEK tubing (Idex 360 µm OD × 150 µm ID, Lake Forest IL, USA) was attached to the PneuWave pump. Isopropanol alcohol was flushed through the PEEK tubing for 5 min for sterilization, then M9 minimal media was flushed through the tubing for 5 min to clear the isopropanol alcohol and prepare the tubing for cells. A magnetic connector lever arm (Fluidic Indexing Probe, CorSolutions, Ithaca NY, USA) and gasket (N-123-03 IDEX, Lake Forest IL, USA) were used to form a compression seal connecting the PEEK tubing to the microfluidic device. M9 minimal media was run through the microfluidic device to pre-wet all of the channels and push out any bubbles.

The tubing and connector were disconnected from the microfluidic device, and cell culture was flushed through the tubing at 60 kPa applied pressure for 5 min. The tubing was then reattached to the microfluidic device, and cells were flowed into the device. Applied pressure was maintained at 60 kPa for the remainder of the experiment. Imaging began two hours after all pressure levels in the microfluidic device were loaded with cells. The two-hour time point was determined from preliminary experiments; the fluorescent signal increased from the start of the experiment until 2 h, then remained constant from 2 to 6 h.

### Microfluidic device hydraulic circuit pressure calculations

The fluidic pressure at the tapered channels within the microfluidic device could not be directly measured, so it was calculated using the pressure at the entry of the device (measured with the PneuWave Pump) and a hydraulic circuit model with the Hagen-Poiseuille law (Eq. 1). *ΔP* is the difference in pressure between upstream end and downstream end of a channel, *Q* is the flow rate, and *R*_*h*_ is the hydraulic resistance of the channel.

1 $${\Delta}{\text{P}}=Q\times {R}_{h}$$

The hydraulic resistance of each linear channel in the microfluidic device was determined individually using either Poiseuille flow (Eqs. 2 and 3) or Plane Poiseuille flow (Eq. 4) where *µ* is fluid viscosity (assumed to be the viscosity of water = 8.9e−4 Pa s), *L* is the length of the channel, *A* is the area of the channel cross-section, *r* is the hydraulic radius, *P* is the perimeter of the channel cross-section, and *H* is the height of the channel. Poiseuille flow (Eqs. 2 and 3) was used for channels where the ratio of the channel cross-section width to channel cross-section height was less than 20, and Plane Poiseuille flow (Eq. 4) was used for channels where the ratio was greater than 20.

2 $${R}_{h}=\frac{8\upmu L}{A{r}^{2}};$$

3 $$r=\frac{2A}{P};$$

4 $${R}_{h}=\frac{12\upmu L}{A{H}^{2}}.$$

The hydraulic resistance of the entire microfluidic device was determined by combining the hydraulic resistance of all of the individual linear channels. Channels in parallel were combined using Eq. (5) where* R*_*Total*_ is the combined hydraulic resistance of channels 1 to n.

5 $$\frac{1}{{R}_{Total}}= \frac{1}{{R}_{1}}+\frac{1}{{R}_{2}}\dots \frac{1}{{R}_{n}}.$$

Segments in series were combined using Eq. (6) where* R*_*Total*_ is the combined hydraulic resistance of channels 1 to n.

6 $${R}_{Total}={R}_{1}+{R}_{2}\dots +{R}_{n}.$$

Due to the complex geometry of the devices, these hydraulic circuit calculations were performed using a custom script in MATLAB (v. 2019a, Mathworks, Natick, MA, USA).

### Hydraulic resistance of tapered channels with cells

When a cell occupies a tapered channel, the cell partially blocks fluid flow and thereby causes an increase in the hydraulic resistance of that channel. Since the fluid flow profile around the cell is irregular (the tapered channel has a trapezoidal cross-section and a cell has a circular cross-section), Eqs. (2) and (3) were not appropriate. To determine the hydraulic resistance of a tapered channel occupied by a cell, we created a model using COMSOL Multiphysics (v 4.3, Stockholm, Sweden). The hydraulic resistance of a tapered channel occupied by a cell is an order of magnitude greater than a tapered channel without a cell. In accordance with experimental observations, it was assumed all tapered channels were occupied by a cell.

### Measuring cell boundary and fluorescence in the microfluidic device

The cell dimensions and cell fluorescence were determined using a custom script in MATLAB (v. 2019a, Mathworks, Natick, MA, USA). The cell dimensions of each cell in the microfluidic devices were determined from the optical transmission image. The optical properties of the channel walls limited the utility of automatic cell detection software necessitating a semi-automated approach. Regions with cells were manually identified (Supplementary Fig. S7); horizontal and vertical line profiles were generated. The midpoint between the peaks and troughs on the line profiles was used to determine the cell boundaries (Supplementary Fig. S7). The distance the cell traveled into the tapered channel was measured as the distance between the cell centroid and the inlet of the tapered channel.

The cell boundary identified from the optical transmission image was mapped to the same location on the fluorescence image. The pixel intensities of all pixels in the cell boundary were summed to calculate the cell fluorescence. All measurements were corrected for the background fluorescence by subtracting the median fluorescence intensity of the entire fluorescence image multiplied by the number of pixels within the cell boundary (Supplementary Fig. S8). The total fluorescence was further normalized according to the experimental imaging parameters. As summarized in Supplementary Table S1, because of the intrinsic protein expression level and the sample background, the imaging parameters during experiments were varied across different strains and mechanical loading methods in order to not saturate the EMCCD camera. Here, all fluorescence measurements were normalized to the imaging parameters of *ΔcrvA* P_murJ_:*msfGFP* in the microfluidic device (integration time t_norm_ = 20 ms, EM_norm_ = 200, and Power_norm_ = 3.6 kW/cm^2^) for comparison. The normalized total fluorescence (FL_norm_) was calculated using Eq. (7):

7 $${\mathrm{FL}}_{\mathrm{norm}}=\mathrm{FL}\left(\frac{{\mathrm{t}}_{\mathrm{norm}}}{{\mathrm{t}}_{\mathrm{int}}}\right)\left(\frac{{\mathrm{EM}}_{\mathrm{norm}}}{\mathrm{EM}}\right)\left(\frac{{\mathrm{Power}}_{\mathrm{norm}}}{\mathrm{Power}}\right).$$

For instance, the background corrected total fluorescence (FL_vxrAB_) calculated from snapshots of *ΔcrvA* P_vxrAB_:*msfGFP* in microfluidic device (tint = 4 ms, EM = 100, and Power = 3.6 kW/cm^2^) was normalized to be:

$${\mathrm{FL}}_{\mathrm{vxrAB}\_\mathrm{norm}}={\mathrm{FL}}_{\mathrm{vxrAB}}\left(\frac{{\mathrm{t}}_{\mathrm{norm}}=20}{{\mathrm{t}}_{\mathrm{int}}=4}\right)\left(\frac{{\mathrm{EM}}_{\mathrm{norm}}=200}{\mathrm{EM}=100}\right)\left(\frac{{\mathrm{Power}}_{\mathrm{norm}}=3.6}{\mathrm{Power}=3.6}\right)=10{\times \mathrm{FL}}_{\mathrm{vxrAB}}.$$

### Measuring cell boundary and fluorescence for hydrostatic pressure and compression experiments

The cell boundary was identified using a custom-written MATLAB software called iQPALM^38^ (https://doi.org/10.6084/m9.figshare.12642617.v1), and in the corresponding fluorescent image, the cell total fluorescence was quantified as the sum of all pixels’ intensity within the cell mask as illustrated in Supplementary Table S3. The average fluorescent background was calculated as the average of 20 random pixels outside the cells.

The average fluorescence of the background was then subtracted from each pixel in the image to produce a corrected image. The total fluorescence of each cell was then found by adding the fluorescence of each pixel in the cell mask of the corrected image. This value was further normalized based on the imaging parameters (Supplementary Table S1) to match the GFP imaging in the microfluidic device as described above.

### Cell sample preparation

Cells were grown overnight (18 h) in LB (Sigma-Aldrich) with appropriate antibiotics at 37 °C with 250 rpm shaking. The overnight culture was diluted in a 1:100 in M9 minimal media supplemented with 8% v/v 50X MEM amino acids (GIBCO) and 4% 100X vitamins (GIBCO), which is referred to as the supplemented M9 in later methods. The cells were grown for 4 h at 37 °C until OD_600_ ~ 0.4 to reach exponential phase then spun down and resuspended in fresh supplemented M9 minimal media.

### IPTG induction

For experiments with isopropyl ß-d-1-thiogalactopyranoside (IPTG) induction, 100 µM of IPTG (Sigma-Aldrich) was added to the cell culture after the 4 h dilution and incubated at 37 °C for another hour before loading cells into the microfluidic device and maintained throughout the entire duration of the experiment.

### Compression experimental protocol

For the compression experiments, the cell overnight culture and dilution were prepared as described in SI Sect. 0. Two milliliters of the 4 h dilution were centrifuged down and resuspended in 30 μL of the supplemented M9. The cells were immobilized for weight application and imaging after being sandwiched between a 3% (w/v) agarose gel and a coverslip. To make the compression sample setup, agarose (Sigma-Aldrich) was dissolved in the supplemented M9 by heating in a microwave, and 50 μL of the molten agarose was dropped onto a glass slide, followed by adding another glass slide which was then removed after the agarose gel solidified at room temperature. Two microliters of the concentrated cell sample were placed onto the agarose gel surface and topped with a coverslip. The supplemented M9 was added on the side to prevent the system from drying, and stainless steel weights were applied on top of the coverslip for 2 h before imaging. We observed that cells at greater applied force experienced greater deformation, as evidenced by greater cell width (Supplementary Fig. S5).

### Hydrostatic pressure experimental protocol

Hydrostatic pressure was applied to a bulk fluid cell culture using a PneuWave Pump (CorSolutions, Ithaca NY, USA). A custom-made plug was used to prevent any flow. Two supplemented M9 cell dilutions were prepared and incubated at 37 °C for 4 h as described in supplementary materials. Meanwhile, 0.03% poly-l-lysine (Sigma-Aldrich) was added to the petri dish and incubated at room temperature for 4 h to functionalize the coverslip. The excess poly-l-lysine solution was washed out with nanopure sterile water after 4 h. One of the supplemented M9 dilutions was then held under hydrostatic pressure for two hours, while the other one was kept under atmospheric pressure. After releasing pressure, 2 mL of cells were immediately centrifuged down, resuspended in 200 μL supplemented M9, and placed 50 μL into one of the compartments of a petri dish (CELLview, glass bottom, four compartments). The same procedure was used for a control cell dilution in which no pressure was applied. After the cell sample was incubated on the petri dish for 3–5 min, the dish was washed with supplemented M9 by pipetting at least 3 times to remove unattached floating cells before adding 200 μL supplemented M9 in each compartment.

### Imaging protocol

To monitor the change of cellular GFP expression level under diverse mechanical stimuli, we used an inverted fluorescence microscope (Olympus IX 71) to illuminate the cells with a 488 nm laser (Coherent INC, Sapphire 488-200 CW CDRH) in wide-field epi mode. A bandpass filter (Semrock, FF01-525/50) was used for the detection of green fluorescence from msfGFP. Due to the difference in intrinsic gene expression level, the illumination pulse duration t_int_, EM gain, and laser local power density varied for the GFP tags on the VxrA promoter and the MurJ promoter to not saturate the EMCDD camera (Andor Technology, DU-897E-CSO-#BV) as listed in Supplementary Table S1. A pi shaper flat top beam shaper (Edmund optics, #12-644) was used to expand the laser beam size in the compression experiments for higher throughput.

### Bacterial cell growth condition and strain construction

Bacterial strains and oligonucleotides used in this study are summarized in Supplementary Tables S2 and S3. All *V. cholerae* strains used in this study were derivatives of *V. cholerae* WT El Tor strain N16961 which were grown on Luria–Bertani (LB) medium with appropriate antibiotics: streptomycin (RPI, cat# S62000), 200 μg ml^−1^; ampicillin (RPI, cat# A40040), 100 μg ml^−1^; carbenicillin (RPI, cat# C46000), 50 μg ml^−1^; Kanamycin (RPI, cat# K22000), 20 μg ml^−1^. For native or IPTG promoter induction, 100 μg ml^−1^ of Benzylpenicillin potassium salt (PenG-Fisher BioReagents, cat# BP914-100) or 100 ~ 500 μM of isopropyl-β-d-1-thiogalactopyranoside (IPTG-GOLDBIO, cat# 12481C50) are supplemented, respectively. Unless indicated otherwise, LB liquid media were inoculated from an overnight culture and incubated at 37 °C with shaking at 200 rpm until reaching mid-exponential phase (OD_600_ ~ 0.4).

*Escherichia coli* DH5α λpir and SM10 λpir were used for all cloning procedures by Isothermal assembly {Gibson Assembly^39^} or conjugal transfer of genes into *Vibrio* strains. Knockout strains were generated by homologous recombination using the suicide vector pCVD442^40^ and amplified PCR products from primer combinations and gene block in Supplementary Table S3. For promoter and *msf*GFP fusion strains, amplified promoter DNAs of *murJ* or* vxrAB* were cloned into pJL1, a suicide vector for chromosomal integration into the *lacZ* locus. For ShyA overexpression strains, ShyA was cloned into pHL100mob using primer pairs for *shyA* (TD-JHS548/549) and the final construct was introduced in a variety of *Vibrio* strains as an episomal copy plasmid with kanamycin resistance gene for plasmid maintenance. The *vxrA*::*vxrA*^H301^^A^ strain construction is described in Ref.^27^, reporters were introduced into this strain as described above, using pJL1.

Although complements of mutants including the P_murJ_:*msfGFP*, P_murJ_^Δ*vxrB* box^:*msfGFP,* vxrA H301A and P_*vxrAB*_*:msfGFP* might also be useful, the three mutants provide sufficient genetic perturbation to test the relationship between mechanical stress and vxrAB signaling while balancing the labor-intensive manufacturing of microfluidic systems for testing under extrusion loading.

### Statistical analysis

Ordinary least squares linear regression models were used to determine trends in the data. When comparing the trends between separate groups, multivariate linear regression was used with the Tukey method for correcting for multiple comparisons. Statistical analyses were performed with a significance level of α = (0.05). Data were analyzed using RStudio.

### Supplementary Information

Supplementary Information.

## Data Availability

The data that support the findings of this study are available within the article and its supplementary material.
