# Supplementary material for: Mechanical stimuli activate gene expression via a cell envelope stress sensing pathway
Source: Sci Rep. 2023 Aug 26;13:13979. doi: 10.1038/s41598-023-40897-w (PMC10460444; doi:10.1038/s41598-023-40897-w)
Supplement: Supplementary file 1 — Supplementary Information. [file 41598_2023_40897_MOESM1_ESM.docx]

Supplementary Materials For:

Title:

Mechanical stimuli activate gene expression via a cell envelope stress sensing pathway

Authors: Christine E. Harper^1,2+^, Wenyao Zhang^3+^, Junsung Lee^1^, Jung-Ho Shin^4,5^, Megan R. Keller^4^, Ellen van Wijngaarden^1^, Emily Chou^1^, Zhaohong Wang^3^, Tobias Dörr^4,5,6*^, Peng Chen^3*^, Christopher J. Hernandez^1*^

^1^Sibley School of Mechanical and Aerospace Engineering, Cornell University, Ithaca, NY 14853, USA

^2^Meinig School of Biomedical Engineering, Cornell University, Ithaca, NY 14853, USA

^3^Department of Chemistry and Chemical Biology, Cornell University, Ithaca, NY 14853, USA

^4^Weill Institute for Cell and Molecular Biology, Cornell University, Ithaca, NY 14853, USA

^5^Department of Microbiology, Cornell University, Ithaca, NY 14853, USA

^6^Cornell Institute of Host-Microbe Interactions and Disease, Cornell University, Ithaca, NY 14853, USA

^+^ C.E.H. and W.Z. contributed equally to this work.

*Corresponding Authors: T.D., [tdoerr@cornell.edu](mailto:tdoerr@cornell.edu); P.C., [pc252@cornell.edu](mailto:pc252@cornell.edu); C.J.H., [christopher.hernandez@ucsf.edu](mailto:cjh275@cornell.edu)

## Supplementary Results

## 1. Analysis of extrusion loading on non-GFP producing cells

To ensure our microfluidic device and imaging arrangement did not inherently introduce a pressure difference-dependent bias in cell fluorescence, we submitted *∆crvA* non-GFP producing cells to the same 2-hour extrusion loading protocol as detailed above (Fig. S1). Cell fluorescence had no pressure difference dependent trend.


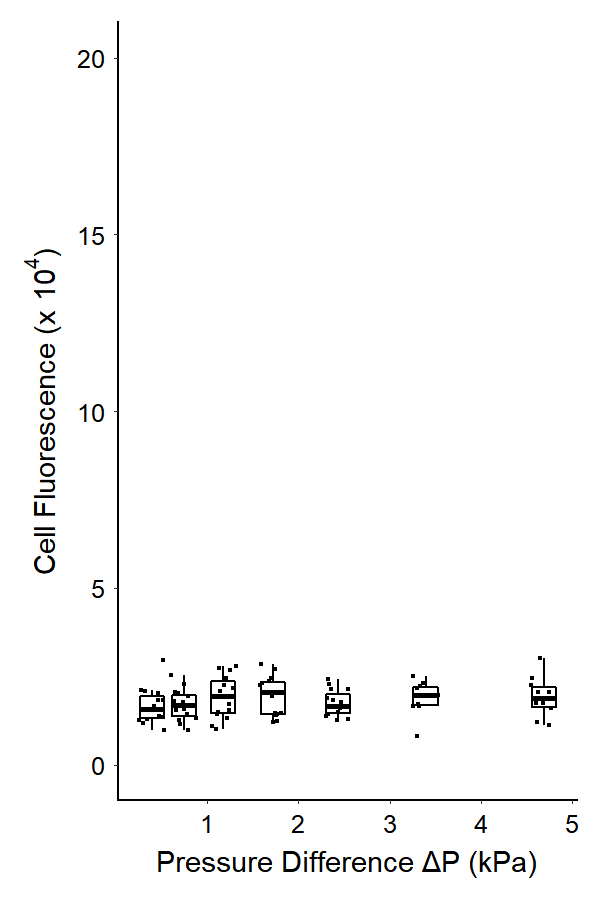


Fig. S1. Cell fluorescence vs pressure difference for *∆crvA* non-GFP producing cells submitted to extrusion loading.

## 1.1 Analysis of extrusion loading on P_murJ_:*msfGFP* cells within 0-30 min of loading

We collected images of the *∆crvA* P_murJ_:*msfGFP* cells within 0-30 min of loading in the microfluidic device as a control to see if there would be a pressure dependent fluorescent trend due to the initial loading procedure. Cell fluorescence had no pressure difference dependent trend for such a short period of mechanical loading (Fig. S2).


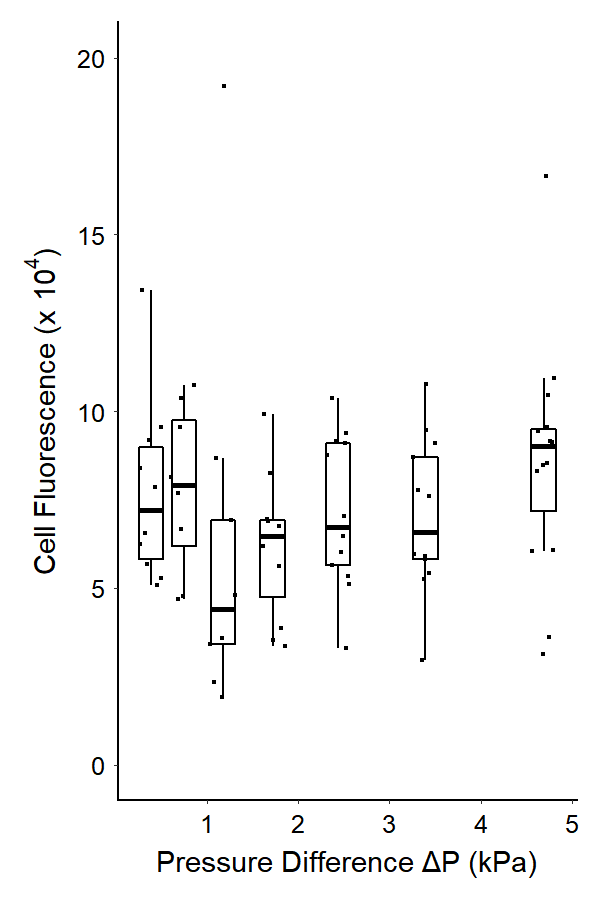


Fig. S2. Cell fluorescence vs pressure difference for ∆*crvA* P_murJ_:*msfGFP* cells imaged within 30 minutes of being loaded into the microfluidic device.

## 1.2 Analysis of extrusion loading on *ΔvxrAB ∆crvA* P_murJ_:*msfGFP* cells

To further investigate if the MurJ expression and GFP production were mediated by VxrAB signaling or another factor, we submitted *ΔvxrAB ∆crvA* P_murJ_:*msfGFP* cells to extrusion loading. Interestingly, *ΔvxrAB ∆crvA* P_murJ_:*msfGFP* cell fluorescence did increase with pressure difference (Fig. S3A). However, *ΔvxrAB ∆crvA* P_murJ_:*msfGFP* cells exhibited a surprising pressure difference-dependent increase in cell volume, not seen in ∆*crvA* P_murJ_:*msfGFP* cells or other strains in the current study (Fig. S3B, D). When cell fluorescence was adjusted for cell volume (Fi. S3C), *∆crvA* P_murJ_:*msfGFP* cells had a significantly greater slope than *ΔvxrAB ∆crvA* P_murJ_:*msfGFP* cells (p<0.05), demonstrating that VxrAB is required for the mechanosensitive increase in cell fluorescence for P_murJ_:*msfGFP* cells.


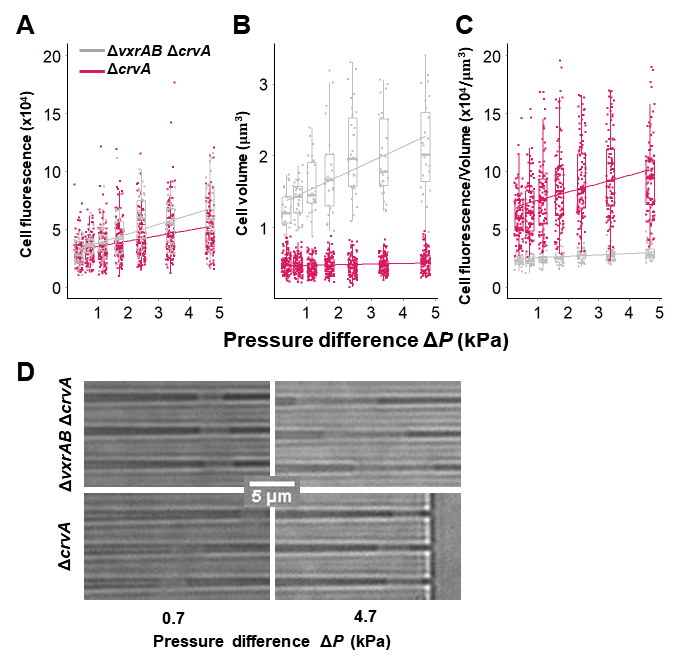


Fig. S3. *ΔvxrAB ∆crvA* P_murJ_:*msfGFP* cells under extrusion loading. (A) Cell fluorescence vs pressure difference for *ΔvxrAB ∆crvA* P_murJ_:*msfGFP* cells indicates a response to magnitude of extrusion loading. However, (B) cell volume vs pressure difference for *ΔvxrAB ∆crvA* P_murJ_:*msfGFP* (black) and *∆crvA* P_murJ_:*msfGFP* (pink) indicates much larger deformations during loading. (C) Cell fluorescence/volume vs pressure difference for *ΔvxrAB ∆crvA* P_murJ_:*msfGFP* and *∆crvA* P_murJ_:*msfGFP* demonstrates that after accounting for the change in cell volume, P_murJ_:*msfGFP* expression is not related to magnitude of extrusion loading. No other strains in this study showed an increase in volume during extrusion loading necessitating this kind of evaluation. (D) Example transmission images of *ΔvxrAB ∆crvA* P_murJ_:*msfGFP* and *∆crvA* P_murJ_:*msfGFP* GFP at 0.7 kPa pressure difference and 4.7 kPa pressure difference.

**Figure S4.** A VxrA H301A mutant phenocopies the *vxrAB* deletion strain in an antibiotic susceptibility assay. Strains were exposed to 100 µg/mL penicillin G (10 X MIC) for 6 hours. Log % survival is colony forming units (cfu/mL) after 6 hours normalized to cfu/mL before addition of antibiotic. Data are averages of at least 4 biological replicates +/- standard deviation.

# Supplementary Figures


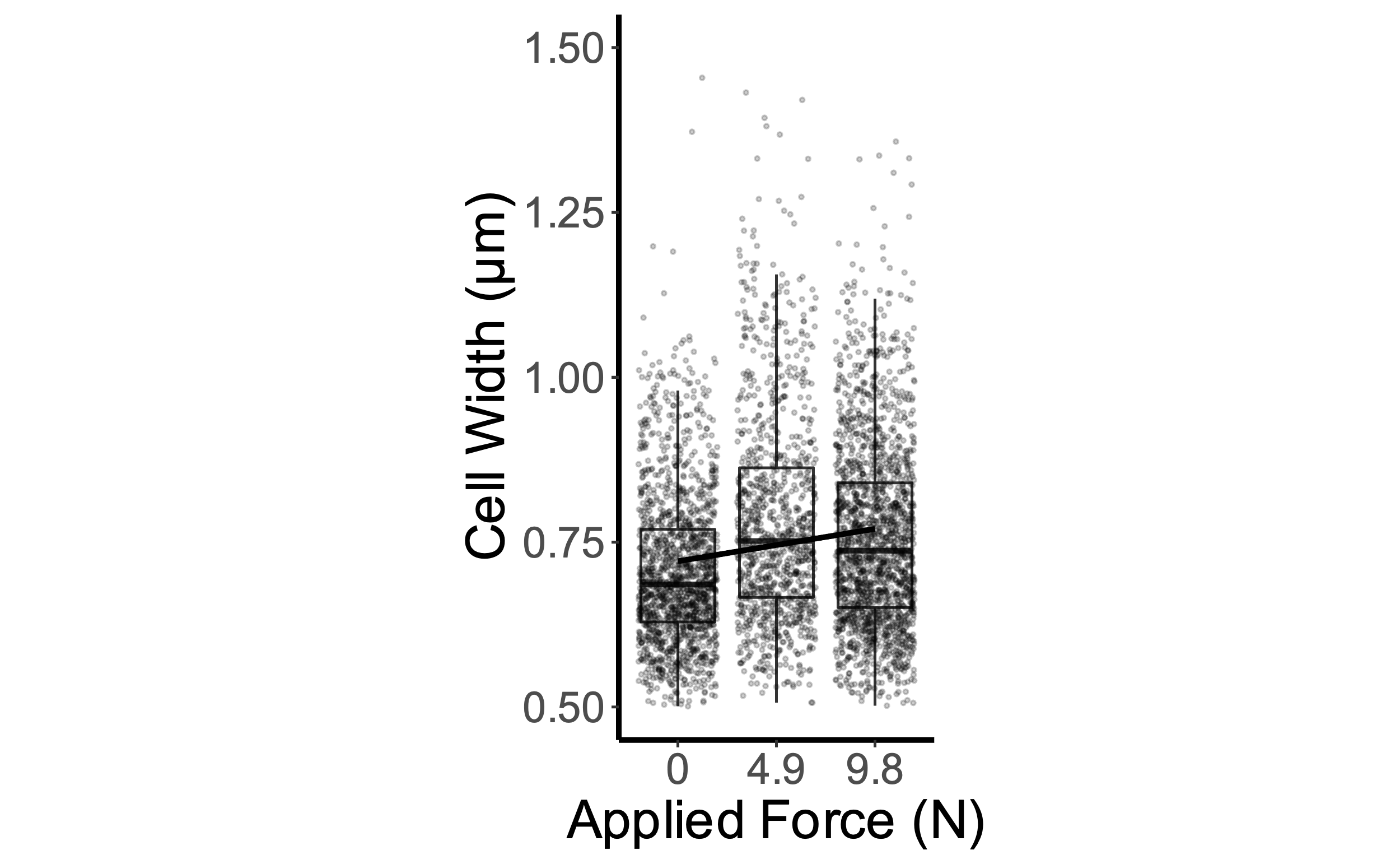


Fig. S5. Cell width (µm) vs applied force (N).


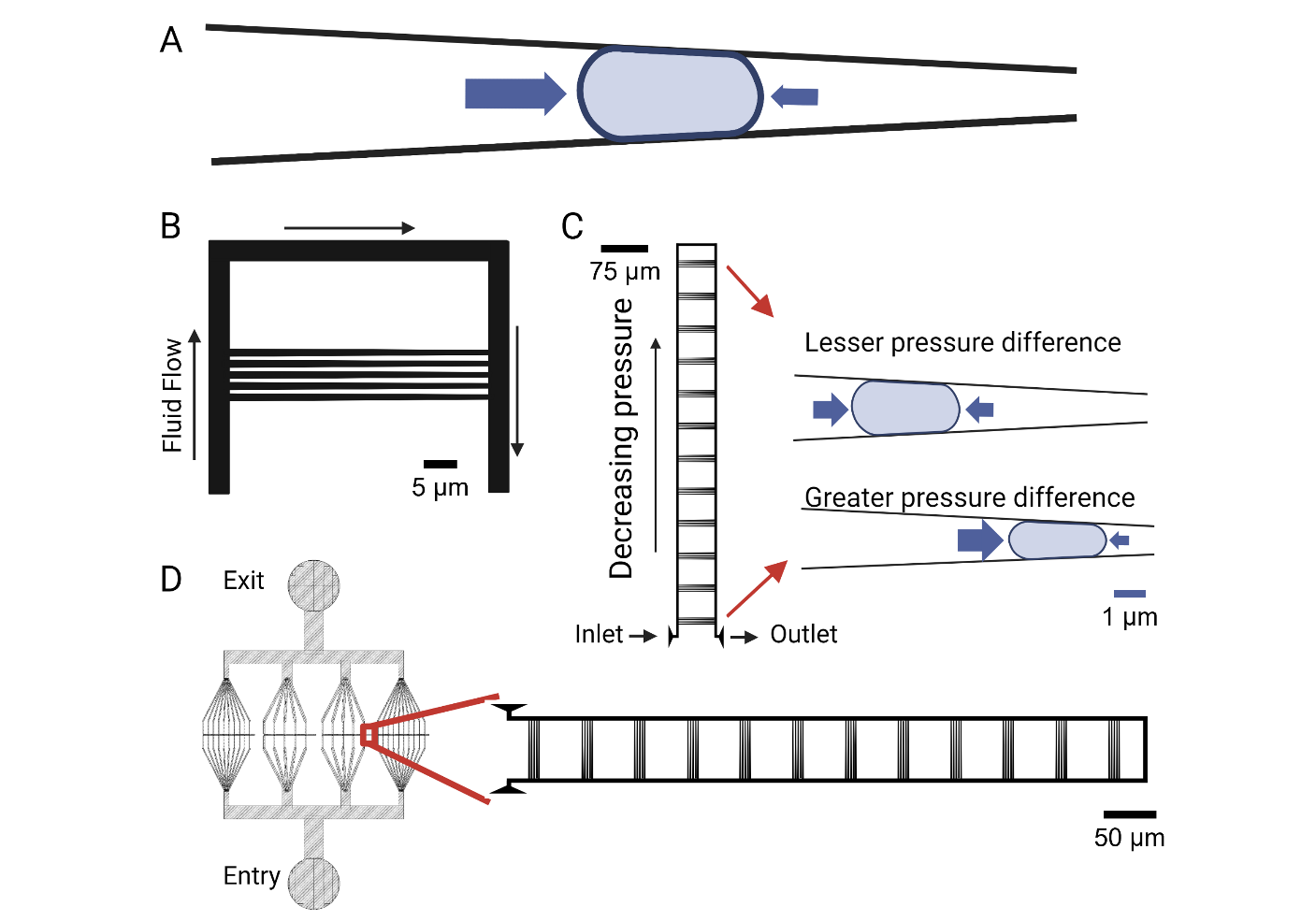


Fig. S6. Extrusion loading microfluidic device to apply controlled mechanical loading to individual cells. (A) A cell forced into a tapered channel. (B) Multiple tapered channels in parallel connected by a bypass channel. (C) A full bypass channel where pressure difference is greatest near the inlet and outlet of the bypass channel. (D) The entire microfluidic device design has multiple bypass channels connected to a single microfluidic device entry port.


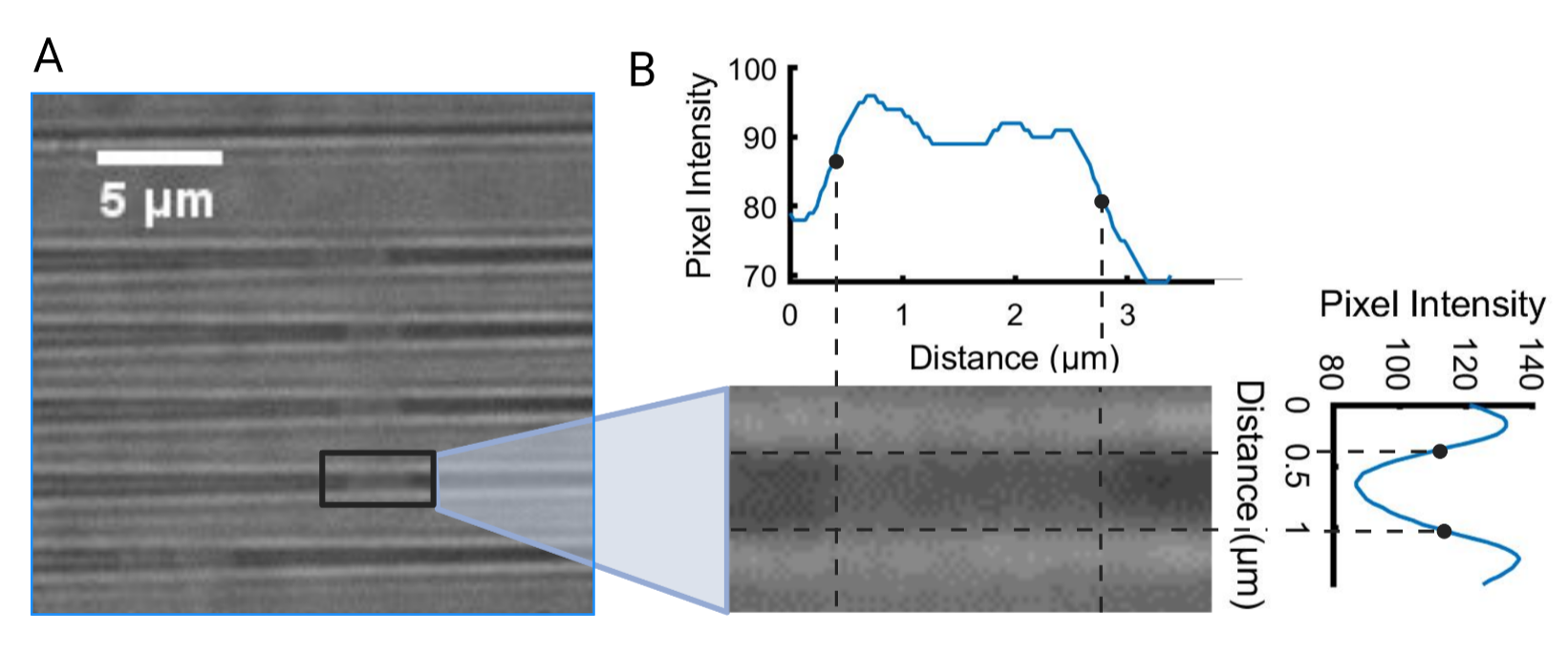


Fig. S7. Cell boundary determination. (A) Transmission image of a cell in the tapered channels of the microfluidic device. (B) Zoomed in region of interest with a cell. Horizontal and vertical line profiles were used to define the cell boundary.


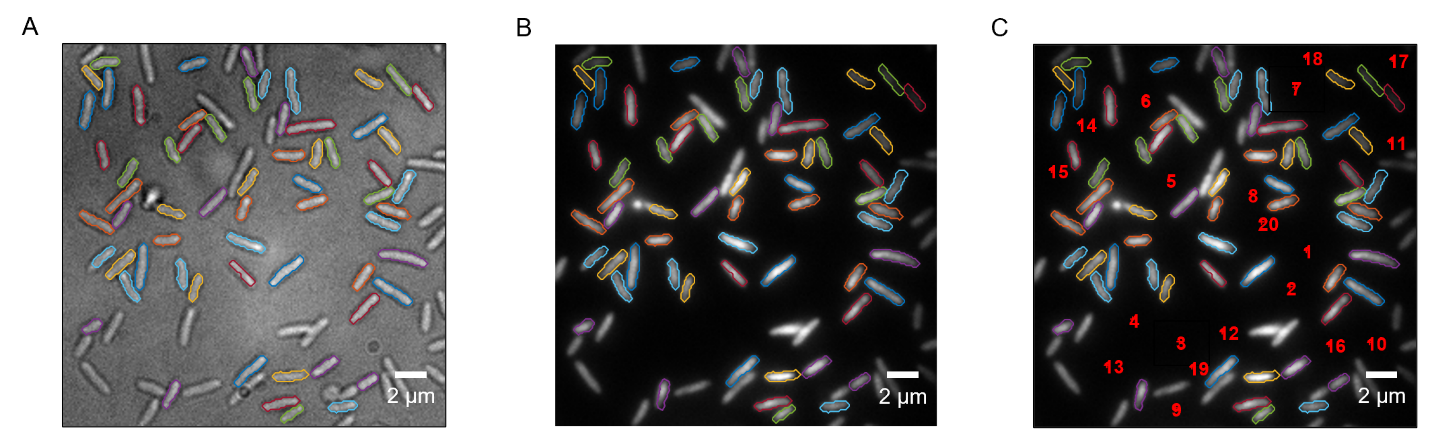


Fig. S8. Illustration of total fluorescence calculation on petri dish A) Transmission image with cell masks overlaid of cell after hydrostatic pressure was applied. B) Fluorescence image with cell masks overlaid. C) Example of average fluorescence image background.

## Supplementary Tables

Table S1. Imaging conditions in this study.

|  | | *ΔcrvA* P_vxrAB_:*msfGFP* | *ΔcrvA* P_murJ_:*msfGFP* | *ΔcrvA* |
| --- | --- | --- | --- | --- |
| t_int_ (ms) | Extrusion Loading | 4 | 20 | 20 |
|  | Hydrostatic Pressure | 4 | 20 | 20 |
|  | Compression | 20 | 20 | - |
| EM gain | Extrusion Loading | 100 | 200 | 200 |
|  | Hydrostatic Pressure | 100 | 150 | 150 |
|  | Compression | 150 | 150 | - |
| 488 power density (kW/cm^2^) | Extrusion Loading | 3.6 | 3.6 | 3.6 |
|  | Hydrostatic Pressure | 3.6 | 3.6 | 3.6 |
|  | Compression | 0.5 | 0.5 | - |

Table S2. Strains used in this study.

| **Strain /plasmid** | | **Relevant description** | **Reference /source** |
| --- | --- | --- | --- |
| ***V. cholerae* strains** | | | |
| N16961 |  | Wild-type O1 Inaba; Str^r^ | Lab stock |
|  | TDW933 | *ΔvxrAB* | ^1^ |
|  | JHS302 | *ΔcrvA* | This study |
|  | JHS615 | *ΔcrvA* P_murJ_*_:_msfGFP* | This study |
|  | JHS623 | *ΔcrvA ΔvxrAB* P_murJ_*_:_msfGFP* | This study |
|  | JHS619 | *ΔcrvA* P_vctPDGC:_*msfGFP* | This study |
|  | JHS626 | *ΔcrvA* *ΔvxrAB* P_vctPDGC:_*msfGFP* | This study |
|  | JHS513 | *ΔcrvA* P_murJ_-*Δ*VxrB Box+*msfGFP* | This study |
|  | JHS514 | *ΔcrvA* *ΔvxrAB* P_murJ_-*Δ*VxrB Box+*msfGFP* | This study |
|  | JHS587 | *ΔcrvA* P_murJ_*_:_msfGFP* +pHL100mob | This study |
|  | JHS595 | *ΔcrvA* P_murJ_*_:_msfGFP* +pHL100mob ShyA | This study |
|  | JHS591 | *ΔcrvA* *ΔvxrAB* P_murJ_*_:_msfGFP* +pHL100mob | This study |
|  | JHS603 | *ΔcrvA ΔvxrAB* P_murJ_*_:_msfGFP* +pHL100mob ShyA | This study |
|  | JHS641 | *ΔcrvA* *ΔshyA* P_murJ_*_:_msfGFP* | This study |
|  | JHS742 | *ΔcrvA ΔshyA* P_murJ_*_:_msfGFP* +pHL100mob | This study |
|  | JHS763 | *ΔcrvA ΔshyA* P_murJ_*_:_msfGFP* +pHL100mob WT ShyA | This study |
| ***E. coli* strains** | | | |
| SM10 λpir |  | Δ(ara-leu)7697 ΔlacX74 ΔphoA PvuII phoR araD139 ahpC galE galK rpsL (DE3) F'[lac+ lacIq pro] gor522::Tn10 trxB pLysSRARE (CamR, StrR, TetR) | Lab stock |
|  | TJES001 | pCVD442 *∆crvA* | This study |
|  |  | pCVD442 *∆vxrAB* | ^1^ |
|  | TJES002 | pCVD442 *∆shyA* | This study |
|  | TJES003 | pJL1 P_murJ_:*msfGFP* | This study |
|  | TJES003 | pJL1 P_vctPDGC:_*msfGFP* | This study |
|  | TJES005 | pJL1 P_murJ_ *ΔVxrB Box*-*msfGFP* | This study |
|  |  | pHL100mob | Lab stock |
|  |  | pHL100mob ShyA | ^2^ |

Table S3. Oligonucleotides and gene blocks used in this study.

|  | **Primer sequence (5’ to 3’)** | **Description** |
| --- | --- | --- |
| **Strain construction** |  | |
| TD-JHS 548 | GAGTTTGACCCGGGTAACAAGAAGTT | ShyA XmaI F |
| TD-JHS 549 | GTTCACTATTGAGCATGCAGAAACGG | ShyA SphI R |
| TD-JHS 550 | GAGTTTGACATGATTAACAAGAAGTTCCT | ShyA Veri New F 2323 |
| TD-JHS 551 | ACGAGTTCACTATTGAGCTGACAGAAACG | ShyA Veri New R 2323 |
| TD-JHS 260 | CATGATTGGCATGGTGGAGC | pJL1 new-F |
| TD-JHS 261 | GTATTGCACAGGACGCGATG | pJL1_new-R |
| TD-JHS 452 | CATGCTGCTTCATGTGATCCGGGTAAC | msfGFP m Rev |
| TD-JHS 028 | CACCATCAGGCAGTTGGCGATCAAGG | VxrA Ver 253TTG |
| TD-JHS 033 | GCGAACTTGCTGCCTTACATGCACATCC | VxrA Ver F1 |
| TD-JHS 034 | GCAGTCTGACAGTAAACCACTCGCG | VxrA Ver F2 |
| TD-JHS428 | CTGAAATGAGCTGTTGACAATTAATC | pHL100 For |
| TD-JHS429 | GCTACTGCCGCCAGGCAAATTCTG | pHL100 Rev |
| TD-JHS 533 | GTAAATCACTGCATAATTCGTGTCGCTCAAG | pHL100 F |
| TD-JHS 571 | CTGTTTGTCTGCGGTGATGTAAACG | msfGFP Ver Rev 500 |
| **Gene block** | GAGTTTGACCCGGGTAACAAGAAGTTCCTTAAAACAAAAGATGAAGTGGAAGTCACTTTTGAATGCGATGCCCCGCAAGCGGCCTCTGAAGTAGCGATTGTGGCCGATTTTTTAGGTTGGCAGCCTGAGCCAATGAAAAAAGTGGCCAAATCAAGCACGTTCAAATTTAAAACTCGTTTGCCGAAAGATCGTGAATTTCAGTTCCGTTATTTGCTCGATAAACAAGAGTGGGTCAATGATCCCCATGCCGATCAGTATATTGCTAACGGTTTTGGTGAAGAAAACTGTTTGTTAACCACCTATCAGTGATTTGGGTAGCATTTTTTGACGAAAGCTCAAAAAATCACAAACAACTAAGTTGCTGCCAGCACACAAAATCCTCTATTTTTGAAAGGCACAAATGTGCCTTTTTTGCTTTAAATTGACGAAAGCGTAGGTGACAAACCGCGCTGAAACCCTTACTCTTGGCCGAGTTTTGACTTTTTTCCACAGGTAAACTGTCTCACATCAACTGTTTTTAGTGGTACCGATTTCTTGAGTATTGCCTTATTTTTGATTGAGGCAAGCGTTGAGAATATGGGCTTTTACCGTTTAGGCTACATTCGTTTCTTTTCTCCAGCGTTCAATCATCACACTCGGTAAATCAGGTCGACTGAAGTAATACCCTTGAATTTGCTCACAGCCCATTTGATAGAGTTTATCCAGTGCTTGTTGGTTCTCTACCCCCTCAGCGACGAGATCGAGTTTAAGCTGGTTAGCAAGCTGAATAATCAACCACACGATACTCTCAGAGGTTTGGTTGGTAAGTAGGTTACGCACAAATGCAGCATCAATCTTGATGCAATCAATCGGATAACTGTGAATGTAGTTAAGGCTCGAATAACCTGTCCCAAAATCATCCAAGGCAATTTTAAAACCCAATTCACGCAATATGGTGAGAATACTGCATACTTCTGCGGCCTTAGAGAGTAAAACCGTTTCTGCATGCTCAATAGTGAAC | *shyA* deletion |
|  | GGGTCTAACTCCCAAAAACTTAGTTCGGTGACATTTTAAGGGCGGGAAATATCCCCGATTTGCTCTTTAATGTCAAATGATTTGTGCAAAAACCCAACTGGACCAAACAAACTTGGTATCGAAGAGCCTTCGCGGTTAAGATGGCGGGGACTTATCTGATCTCACTATGACATTCTTATCCTTTCGAATCAGATAATGTGGTTTTGACTCTTTGTTCGAGGTTACCGTGAGTAAACGCTTATTAAGGAGGAAAGTCACATTCATGATCATGGGAATTCATAAAGGTGAAGAACTGTTCACCGGTGTTGTTCCGATCCTGGTTGAACTGGATGGTGATGTTAACGGCCACAAATTCTCTGTTCGTGGTGAAGGTGAAGGTGATGCAACCAACGGTAAACTGACCCTGAAATTCATCTGCACTACCGGTAAACTGCCGGTTCCATGGCCGACTCTGGTGACTACCCTGACCTATGGTGTTCAGTGTTTTTCTCGTTACCCGGATCACATGAAGCAGCATGATTTCTTCAAATCTGCAATGCCGGAAGGTTATGTACAGGAGCGCACCATTTCTTTCAAAGACGATGGCACCTACAAAACCCGTGCAGAGGTTAAATTTGAAGGTGATACTCTGGTGAACCGTATTGAACTGAAAGGCATTGATTTCAAAGAGGACGGCAACATCCTGGGCCACAAACTGGAATATAACTTCAACTCCCATAACGTTTACATCACCGCAGACAAACAGAAGAACGGTATCAAAGCTAACTTCAAAATTCGCCATAACGTTGAAGACGGTAGCGTACAGCTGGCGGACCACTACCAGCAGAACACTCCGATCGGTGATGGTCCGGTTCTGCTGCCGGATAACCACTACCTGTCCACCCAGTCTAAACTGTCCAAAGACCCGAACGAAAAGCGCGACCACATGGTGCTGCTGGAGTTCGTTACTGCAGCAGGTATCACGCACGGCATGGATGAACTCTACAAATAA | P_murJ_ *ΔVxrB Box*-*msfGFP* |
|  | GGGTCTAACTCCCAAAAACTTAGTTCGGTGACATTTTAAGGGCGGGAAATATCCCCGATTTGCTCTTTAATGTCAAATGATTTGTGCAAAAACCCAACTGGACCAAACAAACTTGGTATCGAAGAGCCTTCGCGGTTAAGATGGCGGGGATTCTATCAGCATTTTTTTACCAATGCTAATACTTATCTGATCTCACTATGACATTCTTATCCTTTCGAATCAGATAATGTGGTTTTGACTCTTTGTTCGAGGTTACCGTGAGTAAACGCTTATTAAGGAGGAAAGTCACATTCATGATCATGGGAATTCATAAAGGTGAAGAACTGTTCACCGGTGTTGTTCCGATCCTGGTTGAACTGGATGGTGATGTTAACGGCCACAAATTCTCTGTTCGTGGTGAAGGTGAAGGTGATGCAACCAACGGTAAACTGACCCTGAAATTCATCTGCACTACCGGTAAACTGCCGGTTCCATGGCCGACTCTGGTGACTACCCTGACCTATGGTGTTCAGTGTTTTTCTCGTTACCCGGATCACATGAAGCAGCATGATTTCTTCAAATCTGCAATGCCGGAAGGTTATGTACAGGAGCGCACCATTTCTTTCAAAGACGATGGCACCTACAAAACCCGTGCAGAGGTTAAATTTGAAGGTGATACTCTGGTGAACCGTATTGAACTGAAAGGCATTGATTTCAAAGAGGACGGCAACATCCTGGGCCACAAACTGGAATATAACTTCAACTCCCATAACGTTTACATCACCGCAGACAAACAGAAGAACGGTATCAAAGCTAACTTCAAAATTCGCCATAACGTTGAAGACGGTAGCGTACAGCTGGCGGACCACTACCAGCAGAACACTCCGATCGGTGATGGTCCGGTTCTGCTGCCGGATAACCACTACCTGTCCACCCAGTCTAAACTGTCCAAAGACCCGAACGAAAAGCGCGACCACATGGTGCTGCTGGAGTTCGTTACTGCAGCAGGTATCACGCACGGCATGGATGAACTCTACAAATAA | P_murJ_:*msfGFP* |

**Supplementary References**

1. Dörr, T. *et al.* A cell wall damage response mediated by a sensor kinase/response regulator pair enables beta-lactam tolerance. *Proc. Natl. Acad. Sci.* **113**, 404–409 (2016).

2. Murphy, S. G. *et al.* Class A Penicillin-Binding Protein-Mediated Cell Wall Synthesis Promotes Structural Integrity during Peptidoglycan Endopeptidase Insufficiency in Vibrio cholerae. *mBio* **12**, e03596-20 (2021).
